# Chloroplast genomes in *Populus* (Salicaceae): comparisons from an intensively sampled genus reveal dynamic patterns of evolution

**DOI:** 10.1038/s41598-021-88160-4

**Published:** 2021-05-04

**Authors:** Jiawei Zhou, Shuo Zhang, Jie Wang, Hongmei Shen, Bin Ai, Wei Gao, Cuijun Zhang, Qili Fei, Daojun Yuan, Zhiqiang Wu, Luke R. Tembrock, Sen Li, Cuihua Gu, Xuezhu Liao

**Affiliations:** 1 National Key Laboratory of Crop Genetic Improvement, Huazhong Agricultural University, Wuhan, 430070 Hubei China; 2 Shenzhen Branch, Guangdong Laboratory for Lingnan Modern Agriculture, Genome Analysis Laboratory of the Ministry of Agriculture, Agricultural Genomics Institute at Shenzhen, Chinese Academy of Agricultural Sciences, Shenzhen, 518120 China; 3 School of Landscape and Architecture, Zhejiang Provincial Key Laboratory of Germplasm Innovation and Utilization for Garden Plants, Key Laboratory of National Forestry and Grassland Administration on Germplasm Innovation and Utilization for Southern Garden Plants, Zhejiang A & F University, Hangzhou, 311300 China; 4 The Second Peoples’s Hospital of Nantong, Nantong, 226000 Jiangsu China; 5 Foshan Green Development Innovation Research Institute, Foshan, 528000 Guangdong China; 6 The College of Horticulture, Shanxi Agricultural University, Taigu, 030801 Shanxi China; 7 Department of Agricultural Biology, Colorado State University, Fort Collins, CO 80523 USA

**Keywords:** Evolutionary genetics, Evolution, Molecular biology, Plant sciences

## Abstract

The chloroplast is one of two organelles containing a separate genome that codes for essential and distinct cellular functions such as photosynthesis. Given the importance of chloroplasts in plant metabolism, the genomic architecture and gene content have been strongly conserved through long periods of time and as such are useful molecular tools for evolutionary inferences. At present, complete chloroplast genomes from over 4000 species have been deposited into publicly accessible databases. Despite the large number of complete chloroplast genomes, comprehensive analyses regarding genome architecture and gene content have not been conducted for many lineages with complete species sampling. In this study, we employed the genus *Populus* to assess how more comprehensively sampled chloroplast genome analyses can be used in understanding chloroplast evolution in a broadly studied lineage of angiosperms. We conducted comparative analyses across *Populus* in order to elucidate variation in key genome features such as genome size, gene number, gene content, repeat type and number, SSR (Simple Sequence Repeat) abundance, and boundary positioning between the four main units of the genome. We found that some genome annotations were variable across the genus owing in part from errors in assembly or data checking and from this provided corrected annotations. We also employed complete chloroplast genomes for phylogenetic analyses including the dating of divergence times throughout the genus. Lastly, we utilized re-sequencing data to describe the variations of pan-chloroplast genomes at the population level for *P*. *euphratica*. The analyses used in this paper provide a blueprint for the types of analyses that can be conducted with publicly available chloroplast genomes as well as methods for building upon existing datasets to improve evolutionary inference.

## Introduction

**Figure 1:**
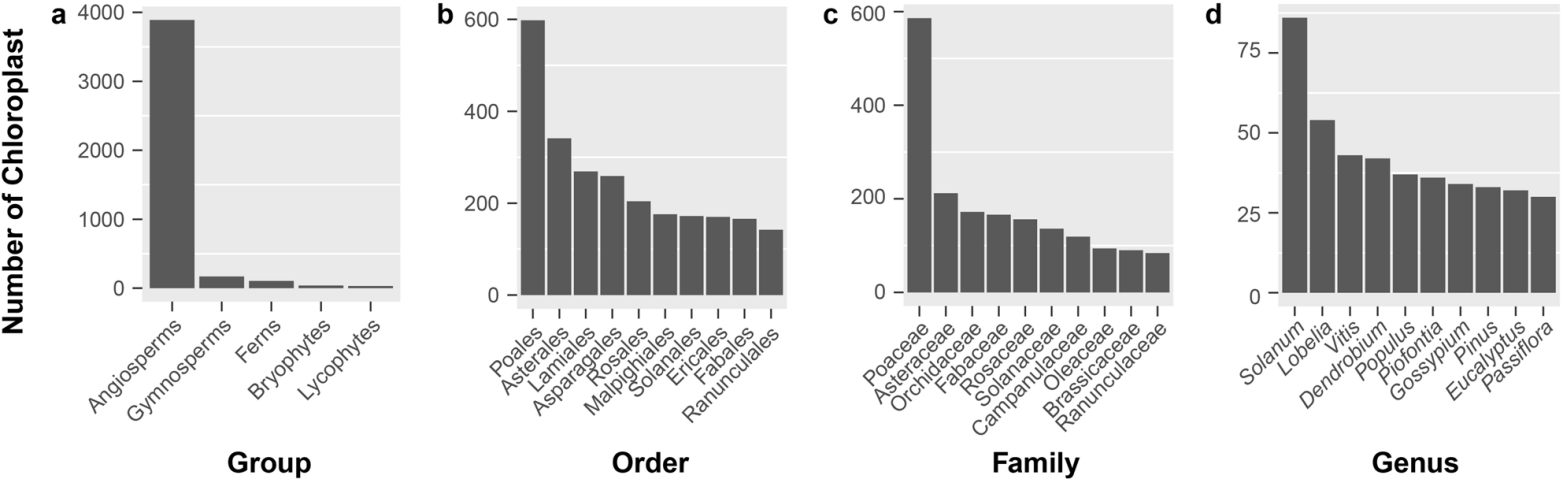
The distribution of finished chloroplast genome at different taxonomic levels. Panels **a**–**d**, represent the number of finished chloroplast genome at group, order, family and genus levels, respectively.

The use of complete chloroplast genomes in studies of plant biodiversity has provided an important advancement over previous methods because of the conserved gene content, mainly uniparental inheritance, and very low rates of recombination helping to reduce problems such as incomplete lineage sorting found among nuclear markers^1, 2^. As with the nucleus and mitochondria, the chloroplast controls essential and specialized cellular functions, mainly photosynthesis, with coding genes retained in this genome distinct to this cellular compartment^3^. The origin of organelle genomes traces back to the incorporation of endosymbiotic cyanobacteria (chloroplast) and alpha-proteobacteria (mitochondria) into proto-eukaryotic host cells about one billion years ago^4–6^. From the time of initial incorporation, organelle genomes have been continually re-shaped during evolutionary history resulting in the current diversity of organelle genomes found throughout eukaryotic lineages today^6, 7^. During this complicated co-evolutionary process, many functional genes or DNA fragments in organelle genomes have been transferred to the host nuclear genome or lost entirely^8, 9^. The outcome of so many gene transfers from organelles to the nucleus is ongoing molecular crosstalk between the different cellular compartments in the form of coordinated cellular signaling and gene expression^10–13^. Despite the coordination of molecular processes between the different genomes, the chloroplast is most often uniparentally inherited during sexual reproduction. As such the finely tuned coordination between the separate genomes can conflict after interspecific hybridization if previously established gene networks are disrupted by the presence of more divergent chloroplast and mitochondrial transcripts interacting with nuclear genes^14, 15^. Such conflicts, if severe enough, can limit the survival of interspecific hybrids providing a means by which species boundaries are maintained in areas of sympatry. Furthermore, changes in ploidy that occur in some cases of interspecific hybridization can also affect genomic interactions through maternally biased gene conversion and paternal homeolog pseudogenization^16^. The size and complexity of chloroplast and mitochondrial genomes are small and streamlined compared with nuclear genomes^17^. Because of this, chloroplast genome sequencing and assembly is relatively straight-forward compared to nuclear genomes. This is evident when comparing the over 5000 complete chloroplast genomes versus the ~ 500 complete nuclear genomes available in the NCBI database (5647 chloroplast/plastid and 538 nuclear genomes as of 15 March 2021). While smaller in size than nuclear genomes, plant mitochondrial genomes are often more complicated than chloroplast genomes owing in part to the multichromosomal structuring and large segments of repetitive DNA resulting in just over 200 complete plant mitochondrial genomes available in the NCBI database^18^. Typically, the chloroplast genome maintains a conserved and quadripartite circular structure across nearly all land plants with sizes ranging from 115 to 165 Kb, and a conserved gene content and gene order^17, 19, 20^. Based on these highly conserved characteristics of plant chloroplast genomes and the development of high-throughput sequencing technologies more species have been sequenced and uploaded to public databases but with varying degrees of annotation quality, and sampling density (Fig. 1). The numerous publicly available chloroplast genomes have provided an essential genetic resource for many types of research including applied studies^21–25^. In traditional molecular systematic and population genetic studies chloroplast markers such as *matK*, *rbcL*, *atpF-atpH*, *trnH-psbA*, and *psbK-psbI* have been recognized as among the best loci for barcoding and tree of life reconstructions^24, 26–28^. Now, that entire genomes can be easily sequenced and assembled, the field of chloroplast phylogenomics has grown in importance^25, 29–31^. In addition, the chloroplast may prove to be the genome of choice for genetic transformation as it occurs in high copy number within the cell, is uniparentally inherited, and is largely non-recombinant^21, 32^. As such well curated chloroplast genome sequence databases with a high density of species and population sampling will be needed for accurate design of transformation constructs.

In this study, we examine the use of chloroplast genomes for inferring phylogenomic relationships, dating inferred divergence times, and identifying the different types of structural changes that have evolved through time using the economically and ecologically important genus *Populus* as a model. Given the importance of *Populus* species, *P. trichocarpa* (black cottonwood) was the first tree species for which the nuclear genome was fully sequenced^33^. Since then, seven additional *Populus* species have been sequenced and finished (https://plabipd.de/plant_genomes_pa.ep). *Populus* is also an important model genus for studying the evolution of dioecy in plants as two different genetic systems of sexual determination have been identified^34, 35^ (XY and ZW systems). Over 30 species are currently recognized in the genus *Populus* with many of the species growing across massive geographic ranges^36, 37^. At present, over 40 available chloroplast genomes have been sequenced, representing most named species (including several hybrid species) from *Populus*. However, in regard to taxonomy it should be noted that 98 species level designations/names are currently accepted for *Populus* species of which 26 are used to denote hybrid taxa and an additional 466 species names are either synonymized or unresolved (http://www.theplantlist.org/tpl1.1/search?q=populus). Furthermore, 18 additional names are accepted at the infraspecific rank within 13 *Populus* species. Such taxonomic conflict underlies the problem classic systematists have had using morphology alone in properly characterizing a genus with wide-ranging morphological characters^37^, rampant hybridization^36, 38, 39^ and extremely long-lived individuals overlapping with recent generations^40, 41^—all potentially obscuring signals of divergence. As such genetic markers that provide robust signals of divergence are needed to accurately reconstruct the history of cladogenesis in *Populus*. The genus *Populus* is an ideal study system for examining the utility of chloroplast genomes in inferring evolutionary history and patterns in trees with near comprehensive species sampling as well as population level sampling in some species. We will explore the following questions: 1) How is chloroplast genome structure conserved across the genus? 2) What types of mutational changes have accumulated during cladogenesis and are these lineage specific? and 3) What other evolutionary patterns can be observed from comprehensive chloroplast data sets?

## Results

### The sequencing progress of land plant chloroplast genomes as a function of the percentage of total species diversity in a clade

Before the development of modern sequencing technologies, researchers often employed one or several genetic markers to investigate pertinent biological questions^24, 28^, and chloroplast markers were among the main genetic resources in those days. To date, over 4000 complete chloroplast genomes (by the date of 03/15/2021, there are 5647 chloroplast/plastid of non-redundant species) have been deposited in the NCBI database^18^ (Fig. 1, table S1). To illustrate the phylogenetic distribution of the finished chloroplast genomes, we will focus this part mainly on the land plants and separate them into different groups in Fig. 1.

The angiosperms are the most specious group of land plants with well over 300,000 species^42^ and with the greatest number of completed chloroplast genome sequences. However, because the angiosperms are so diverse the 4235 completed chloroplast genomes of non-redundant species (data collected on 02/24/2020 from NCBI database, including 4210 chloroplast/plastid accessions starting with “NC_” from https://www.ncbi.nlm.nih.gov/genome/browse#!/organelles/, and 25 other accessions, Table S1) account for only about 1.3 percent of the total number of species, whereas the ~ 100 complete chloroplast genomes from the gymnosperm accounts for about 15 percent of the total species (Fig. 1). This trend is also true for completed plant mitochondrial genomes. However, the number of finished plant mitochondrial genomes are just over 200^18^. While angiosperms are the most diverse group of land plants and are important for providing much of humanities needs for survival, groups such as ferns (105 complete chloroplast genomes accounting for 1% of all fern species), lycophytes (32; 2.5%), bryophytes (39; 0.2%), and even algae should have more chloroplast genomes sequenced in order to gain a more comprehensive understanding of chloroplast evolution.

At the order level (Table S1), the top orders for number of completed chloroplast genomes are from Poales (598; 3.2%), Asterales (341; 1.3%), Lamiales (269; 1.1%), Asparagales (259; 0.7%), Rosales (204; 2.5%), Malpighiales (176; 1.1%), Solanales (172; 4.1%), Ericales (170; 1.4%), Fabales (166; 0.6%), Ranunculales (142; 3.1%), and Pinales (139; 60.2%). At the family level, the top families for number of completed chloroplast genomes are Poaceae (586; 5.2%), Asteraceae (212; 0.8%), Orchidaceae (172; 0.7%), Fabaceae (166; 0.8%), Rosaceae (156; 5.6%), Solanaceae (136; 5.5%), Campanulaceae (119; 5.0%), Oleaceae (94; 15.3%), Brassicaceae (90; 2.3%), and Ranunculaceae (84; 3.3%). At the genus level, the top ten most sequenced genera for chloroplast genomes are *Solanum* (86; 6.1%), *Lobelia* (54; 13.0%), *Vitis* (43; 57.3%), *Dendrobium* (42; 2.5%), *Populus* (39; ~ 100.0%), *Piofontia* (36; 57.1%), *Gossypium* (34: 68.0%), *Pinus* (33; 29.2%), *Eucalyptus* (32; 4.0%), and *Passiflora* (30; 4.8%). From these numbers, it is clear that sampling effort has been unevenly distributed among different plant groups/genera. The target species or families for chloroplast sequencing have been those with high economic values (e.g. crop and ornamental species). However, this sampling approach does not result in even sampling based on phylogenetic distance that would result in more useful data for overall inferences on chloroplast evolution. Genera such as *Vitis* and *Populus* are approaching comprehensive levels of sampling and provide excellent study systems for understanding chloroplast evolution in greater resolution.

One of the main reasons behind the rapid increase of finished chloroplast genomes is due to the recent development of sequencing technologies, such as next generation sequencing (NGS), third generation sequencing (TGS), and the decrease in cost associated with these sequencing methods. Before the development of NGS, the traditional Sanger sequencing method was the dominant process for sequencing chloroplast genomes^17^. Between 1986 and 2015, only 400 complete chloroplast genomes had been published using Sanger sequencing^17^, from 2015 to now the number increased by nearly ten times^18^. As such the rate of chloroplast genome sequencing in the past 5 years has increased nine times over the previous twenty 20 years (~ 3600 genomes from 2015–2020/ ~ 400 genomes from 1986 to 2015 = 9 times). In addition to the improvements in sequencing technology, bioinformatic methods for annotation and assembly have also improved making post sequencing processing more efficient and accurate^43^. In addition to speed and accuracy of NGS sequencing the huge reductions in cost have also contributed to the increase of completed chloroplast genomes. With the massive increase in publicly available chloroplast genome sequences, questions regarding data quality, sampling density, and appropriate analytical methods for inferring evolution with large, differentially sampled datasets, become increasingly important. As such we employed chloroplast genomes from the genus *Populus* to assess issues of data quality and analytical methods in a lineage of plants that have been nearly comprehensively sampled for all species and in at least one instance have population level sampling.

### The completeness of chloroplast genomes in *Populus*

For the 39 sequenced *Populus* chloroplast genomes, the full-length variation is approximately 3.5 Kb (155,096–158,591 bp). This level of variability in length is similar for the inferred age of *Populus* (~ 48 mya)^44^ when compared to other clades with extensive chloroplast genome sequencing like Solanaceae (~ 55 mya)^45^, but differ greatly in regard to the number of species (*Solanum* =  ~ 2400 vs *Populus* =  ~ 90). As such length variability of chloroplast genomes may be more constrained by time than the number of speciation events in a clade, as is expected for a genome that is essentially nonrecombinant and highly constrained in function. However, differences in genome content between genera should be compared to see if regions of variability are generalizable between distantly related lineage.

In regard to the publicly available chloroplast genomes in *Populus*, seven species were found to possess between 1 and 40 N bases in their published genomes. These errors likely arose from sequencing or assembly errors in repetitive regions such as poly A/T regions. The 40 N bases in *P. yunnanensis* (NC_037421; isolate MaoKS-CX-2014-270) were located in the intergenic region of *psaA-ycf3*. None of the other 38 chloroplast genomes contained errors at this site suggesting that the *P. yunnanensis* sequence was not properly checked for deletions or polymorphisms but could be easily updated with resequencing. Such discrepancies point to the need for these genomes containing Ns to be re-sequenced (possibly with updated TGS methods) and/or reassembled to improve inferences made with this data. Using incomplete genomes can reduce the accuracy of inferring relationships among species^17^.

In addition to problems with nucleotide calls in the *Populus* chloroplast genomes, several gene annotations were inconsistent regarding number and content of genes. In general, because gene content is so consistently conserved in chloroplast genomes^29^, it is rare to see gene duplications or deletions at the genus or even family level^30, 46^. As such we reannotated all 39 chloroplast genomes in *Populus* by incorporating all gene content to improve the continuity of annotations across the genus (Supplement Table S2). The following issue were found with annotations: *P. pruinosa* (NC_037417) lacked annotations for both copies (in each inverted repeat region) of *rps7, P. ilicifolia* (NC_031371) and *P. davidiana* x *P. alba* (NC_044462) had only a single *rps7* copy annotated, similarly *P. balsamifera* (NC_024735), had only one copy of both *ndhB* and *ycf2* where two should be annotated (one in each IR), and lastly the previous annotation of *ycf1* in *P. pruinosa* (NC_037417) and *P. szechuanica* (NC_037419) were removed. Given the above results, researchers should practice caution both when using NCBI data and when uploading completed chloroplast genomes. It should also be noted that annotations are improved when sampling density within a lineage is increased and thus periodic lineage-based reannotations should be conducted for groups like *Populus* that have comprehensive sampling.

### Phylogenetic analyses of *Populus* chloroplast genomes

**Figure 2:**
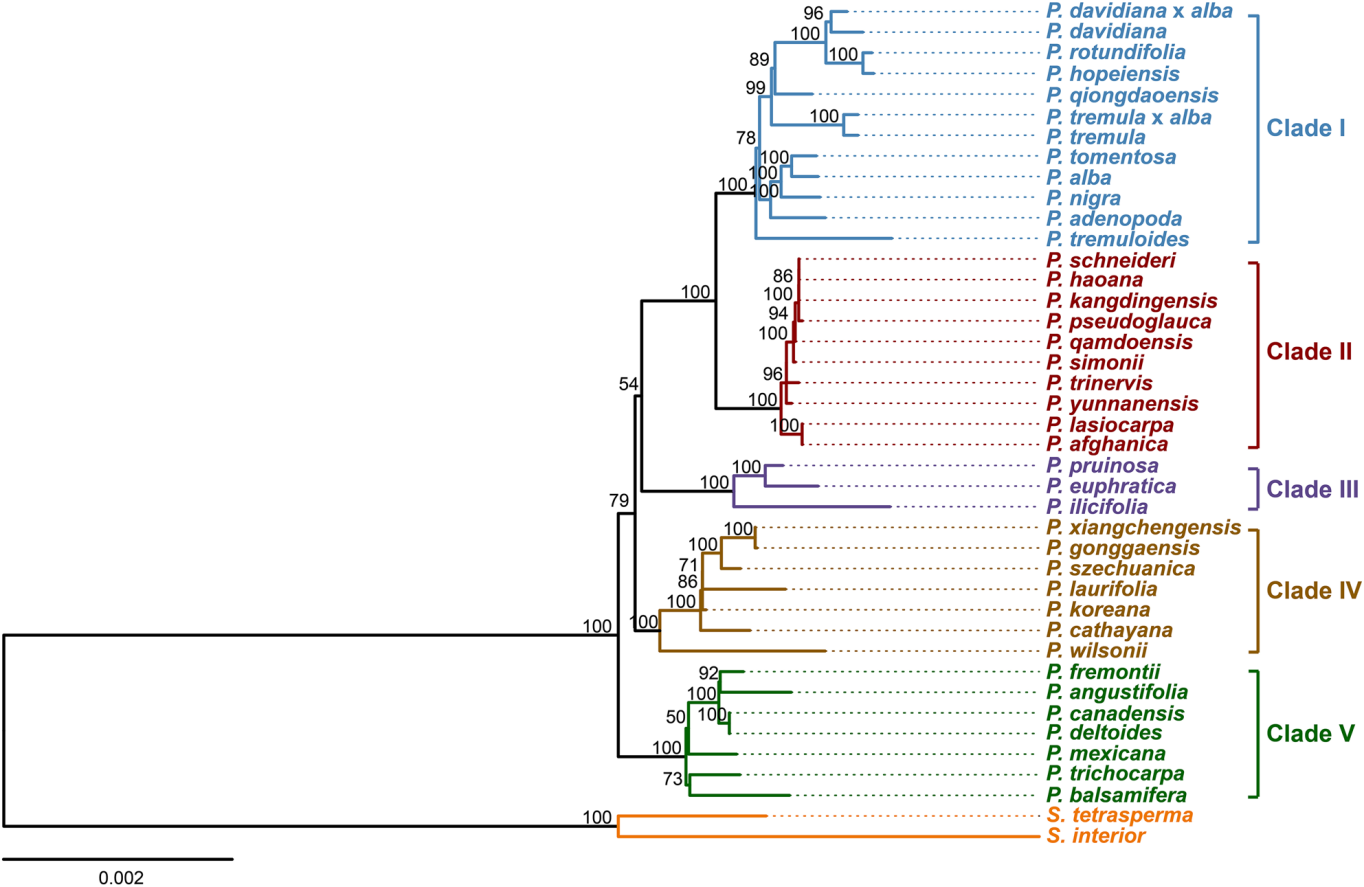
Phylogenic tree of 39 *Populus* species based on an alignment of 76 chloroplast coding genes using the ML method. Two *Salix* species were used as out-groups. The number above each node indicates the BS support value for that clade.

Chloroplast genomes have become an indispensable tool in resolving plant phylogenetic relationships given the relative ease in generating complete high-quality sequences and the lack of recombination^46, 47^. By employing all coding genes and the full length of the chloroplast genomes from 39 *Populus* species and two out group species from *Salix*, the phylogenetic tree for *Populus* using maximum likelihood^48^ (Fig. 2) was analyzed.

Five sub-clades were resolved in the analyses each with high support values (referred to with roman numerals I-V; Fig. 2). Membership of each clade is nearly identical to the results in Zong et al.^37^ save the samples added herein and differences with internal branching order. The species *P. szechuanica* and *P. trinervis* resolved in different clades and may reflect mislabeling or misidentification of species in GenBank. For instance, as we employed *P. szechuanica* NC_037419 and Zong et al.^37^ employed *P. szechuanica* MK267303 suggesting one of these samples might be mislabeled or if biological in origin could reflect differences in parentage in past hybridization events. Branching order above the level of the recognized subclades also differed between our analysis and Zong et al.^37^.

When comparing the cp tree to the tree using nuclear derived SNVs (single nucleotide variants) in Wang et al.^36^ the topology and membership of well supported clades differs. For example, *P. fremontii*, *P. deltoides*, and *P. nigra* of the section Aigeiros were in a late diverging clade with species from sections Aigieros, Tacamahaca, and Leucoides in the nuclear tree whereas in the cp tree *P. fremontii*, and *P. deltoides* are in an early diverging clade (clade V) and *P. nigra* resolved in a late diverging clade (clade I) with species from the section Populus/Leuce. Consensus among the nuclear SNV data the cp tree of Zong et al.^37^ and the cp genome analyses conducted here is found in the section Turanga containing all the same species in the same branching order. The phylogenetic trees from either of the two genomes (cp or nuclear) also demonstrated that polyphyly and paraphyly are common among the classic delimitations of most sections of *Populus*. For instance, species of section Aigeiros were found in clades I, II, and V of our analyses and were polyphyletic in a late diverging clade in Wang et al.^36^. These results reflect what is already well known for *Populus* that hybrid speciation and incomplete lineage sorting resolve conflicting topologies when genomes from different cellular components are used in phylogenetic analyses^37, 49^. For this current study, we focused on comparisons between the five well supported cp clades of *Populus* (Fig. 2) as a basis for understanding chloroplast evolution and compare this to nuclear phylogenies and taxonomic designations where applicable.

### Repeat content in *Populus* chloroplast genomes

**Figure 3:**
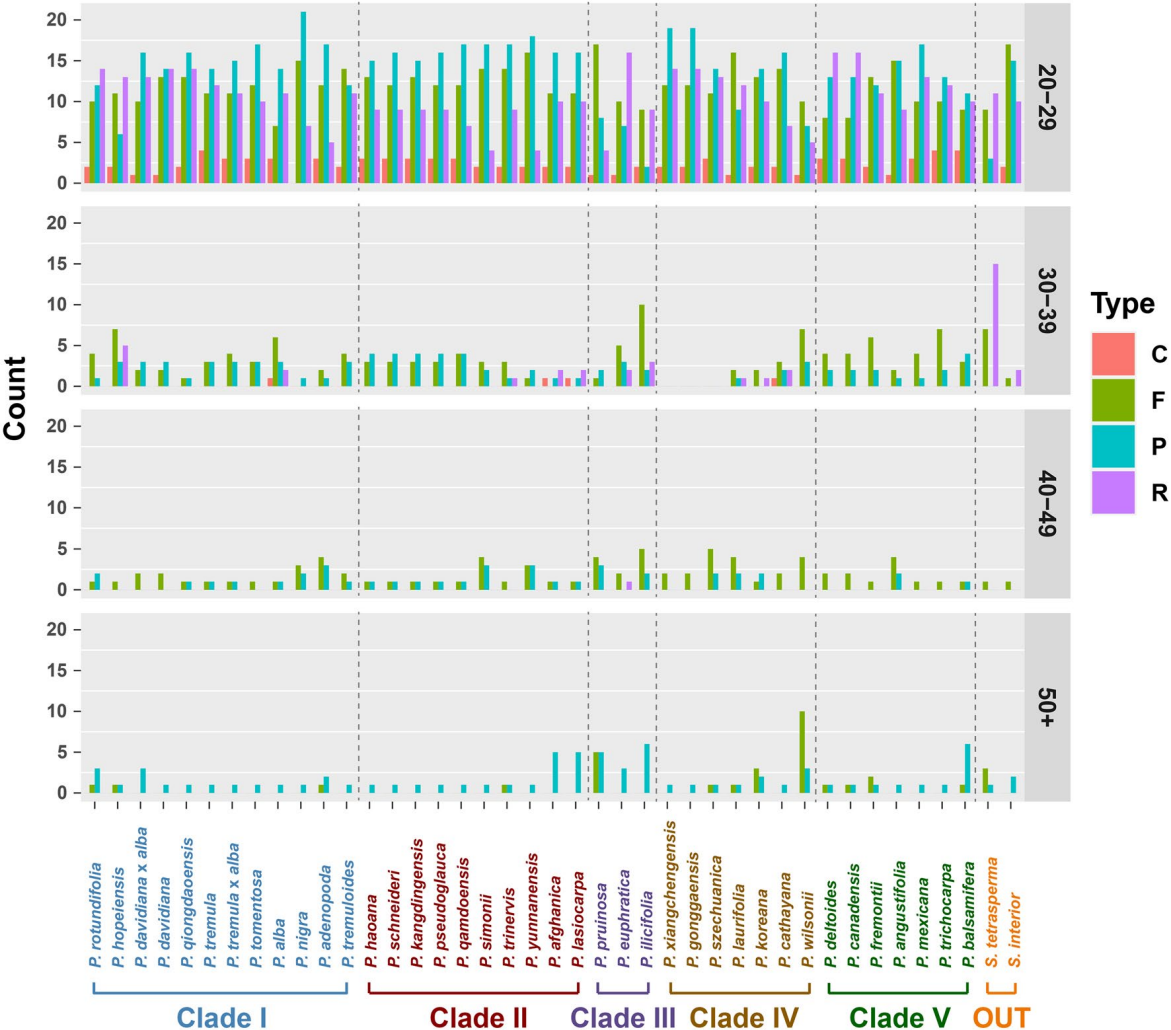
Variation of repeat abundance and type within chloroplasts from 39 *Populus* species and two outgroups.

Repeat content is important for chloroplast genomic comparisons as they can vary between different lineages and lead to differences in genome size and structural rearrangements^46, 50^. To compare the number and content of small repeats (mostly ranging from 20 to100 bp) in chloroplast genomes of *Populus*, we systematically analyzed the variation across the 39 species and within the five clades to see if any patterns were present between lineages. The repeats were identified using REPuter (https://bibiserv.cebitec.uni-bielefeld.de/reputer) and classified by motif type to include direct forward (F), reverse (R), complement (C), and palindromic matchs (P) (Fig. 3). The greatest number of repeats were from the smallest size class (20–29 bp; 77.1%, 1504 out of 1950) in *Populus*. The number of repeats from the longer size classes (> 30 bp) were five or less per species for any given motif. The trend of a greater abundance of short repeats follows that for other chloroplast genomes and is the inverse of plant mitochondrial genomes wherein longer repeats are more abundant^6^.

The least common repeat type across all size classes were complement (C) repeats with no more than five found in any given species. The palindromic repeats (P) were the most abundant type (36.3%, 546 out of 1504) in the 20–29 bp group with forward (F) and reverse (R) repeats the second most abundant (30.9% and 27.0% respectively). Variation between and within clades is clearly apparent especially among the larger repeat size classes. For instance, clade III has the lowest number of palindromic repeats (P) in the 20–29 bp, but the only reverse (R) repeats in 40–49 bp size class. In the 30–39 bp group the reverse (R) repeats are absent in clade IV, while in clade III and clade V the complement (C) repeats were absent. Within clades repat abundance varied such as in clade IV, were *P. wilsonii* (NC_037223) which had the lowest number of repeats in 20–29 bp group (23) while in the same clade, *P. xiangchengensis* (NC_040953) and *P. gonggaensis* (NC_040873) both had 24 more (47 total). However, in 30–39 bp group, *P. xiangchengensis* (NC_040953) and *P. gonggaensis* (NC_040873) had zero repeats, and *P. wilsonii* (NC_037223) had highest number in this size class (7). In 40–49 bp size class, repeat numbers were low and fairly consistent across clades. In 50 + bp group, *P. wilsonii* (NC_037223) had highest number of repeats of all 39 species sampled. From comparisons of repeat number across clades and species it appears that repeat number and type vary suggesting that there may have been differences in transposition activity and/or mutations (making the repeats undetectable) at both the species and clade levels.

### Simple sequence repeat abundance in *Populus*

**Figure 4:**
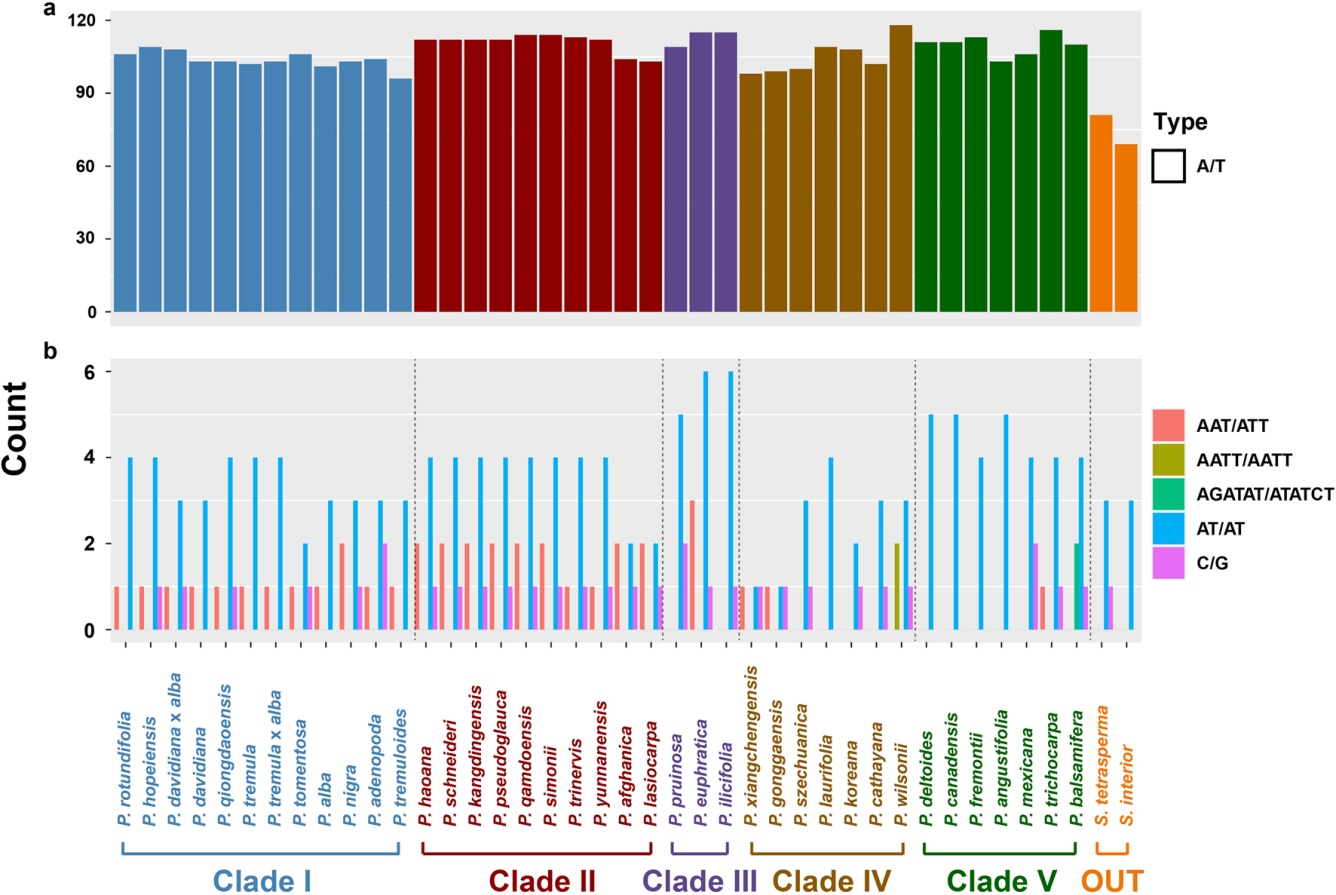
The number of Simple Sequence Repeats (SSRs) in *Populus* chloroplast genomes and two out-group species. a: A/T SSRs; b: other common SSR motifs.

Because of high mutation rates simple sequence repeats (SSRs) are important loci in population genetic, phylogenetic and biogeographic studies^51^. To better characterize the distribution of SSRs in the *Populus* genus, we conducted several analyses to detect and compare SSRs. The most common SSR type detected were homopolymer A/T repeats accounting for 95.2% on average of all SSRs in *Populus* (Fig. 4a). The high abundance of this type of SSR is in line with most other chloroplast genomes studied thus far^46^. By contrast only one or two polycytosine (poly C)/polyguanine (poly G) SSRs (Fig. 4b) were detected per genome. The length variation of poly A/T SSRs was from 10 to 31 bp, with the number of poly A/T SSRs varying from 96 to 118 across the *Populus* chloroplast genomes. The two outgroup species from *Salix* contained far fewer poly A/T SSRs than all other *Populus* species when applying the 10 bp cut-off for homopolymer SSR detection. As poly A repeats in cpDNA can be associated with modulating transcription the differences between *Salix* and *Populus* A/T repeat abundance may reflect differences in transcriptional modulation between the two lineages^52^. However, more in-depth analyses from *Salix* should be conducted to address this question.

The abundance of the most common SSR repeat motifs are summarized in Fig. 4b. The dinucleotide repeat AT_x_ was the most abundant dinucleotide SSR, with numbers varying from 1 to 6 per chloroplast genome. Clade III and clade V had the most dinucleotide repeats with four or more in all species. In clade IV, some species had one and others had four. The most common trinucleotide repeats AAT_x_ varied from zero to three per genome with clades IV, V, and the outgroup with numerous species that did not contain this repeat. The tetranucleotide AATT_x_ SSR was only found in *P. wilsonii* and the hexanucleotide AGATAT_x_ was only found in *P. balsamifera*.

### Sequence diversity in *Populus*

**Figure 5:**
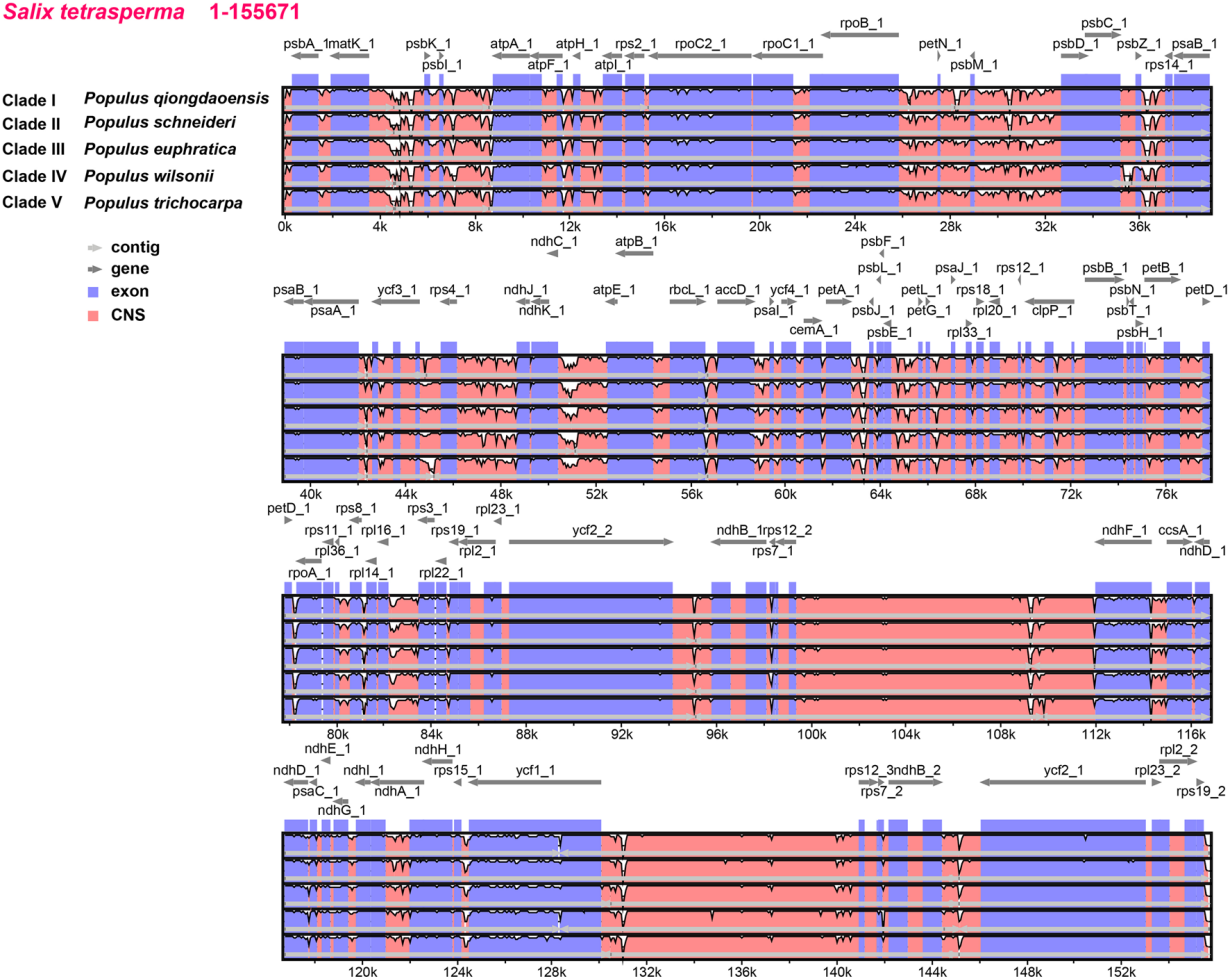
Global alignment of four *Populus* chloroplast genomes from different clades using mVISTA with *Salix interior* as reference. Y-axis shows the range of sequence identity (50–100%). tRNA and rRNA genes were not present in this figure. Numbers 1 and 2 after gene names indicate duplicate copies.

By employing the mVISTA tool, we analyzed the global sequence diversity in *Populus* using one representative species from each of the five clades (Fig. 5). From this, it is clear that the five sampled *Populus* chloroplast genomes exhibited high sequence similarity with some notable exceptions such as the non-coding region between *psbC_1* and *psbZ_1* in *P. wilsonii*. High levels of sequence similarity across chloroplast genomes at this phylogenetic depth is common among land plants^30, 46^. At the genome level, the large single copy region (from the start position to *rpl22*) had the most variable positions and the two inverted repeat regions (*rpl22* to *ndhF* and *ycf1* to the *rpl22*) had the fewest variable positions.

In order to further characterize sequence variation across all *Populus* species, we conducted sequence variation comparisons on a data set partitioned into coding and non-coding sets. Sequence variation was assessed using the T-Coffee score^53^ to report the variation in the alignment of all species (Figure S1). From this analysis most parts of the alignment scored above 950 suggesting high levels of similarity across all genomes. Using a score of 925 as a cut-off, 15 sites were identified as highly variable. Region *atpA*-*atpF* contained the lowest score at 750 with the second lowest score (870) in *ndhD* − *psaC* from the small single copy. All 15 highly variable sites were located in non-coding sequences of the genome. One of the 15 highly variable sites was from the inverted repeat region (*rps19*-*rpl2*), two sites were from the small single copy region (*ccsA-ndhD*, *ndhD-psaC*), and the remaining 12 sites were in the large single copy region.

**Figure 6:**
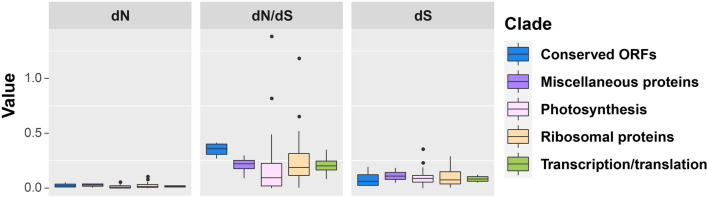
The mode and strength of selection of 75 chloroplast protein coding genes in *Populus*.

To detect proteins undergoing selection in any of the 76 chloroplast protein coding genes, we calculated the dN (nonsynonymous substitution rates), dS (synonymous substitution rates) and the ratio of dN/dS (quantifying the strength of selection) (Fig. 6) while employing the above phylogenetic tree (Fig. 2) as a basis of comparison. The 75 genes (*psaI* omitted) were divided into five groups based on function and included photosynthesis genes (44), ribosomal proteins (19), transcription/translation genes (4), conserved ORF genes (4), and miscellaneous proteins (4). The dN average values and variability around the average across all five gene categories was very low throughout *Populus* with outliers in the photosynthesis and ribosomal protein gene groups respectively. The dS values were somewhat higher than the dN values but relatively low with little variability save the outliers in the photosynthesis gene group (Fig. 6). The dN/dS values indicate that nearly all genes are undergoing strong purifying selection which is expected for genes under strong functional constraints like those found in most photosynthetic plants. However, genes *psbJ* and *rpl2* were outliers with high dN/dS values suggesting that those (photosynthetic and ribosomal protein genes respectively) genes are undergoing positive selection. The other two genes *petL* and *rps8* (also photosynthetic and ribosomal proteins) were the other outliers in dN/dS values. Aside from those outliers the photosynthetic genes had the lowest dN/dS values indicating the function of these genes are highly constrained.

### Dynamics of junction boundaries in *Populus* chloroplast genomes

**Figure 7:**
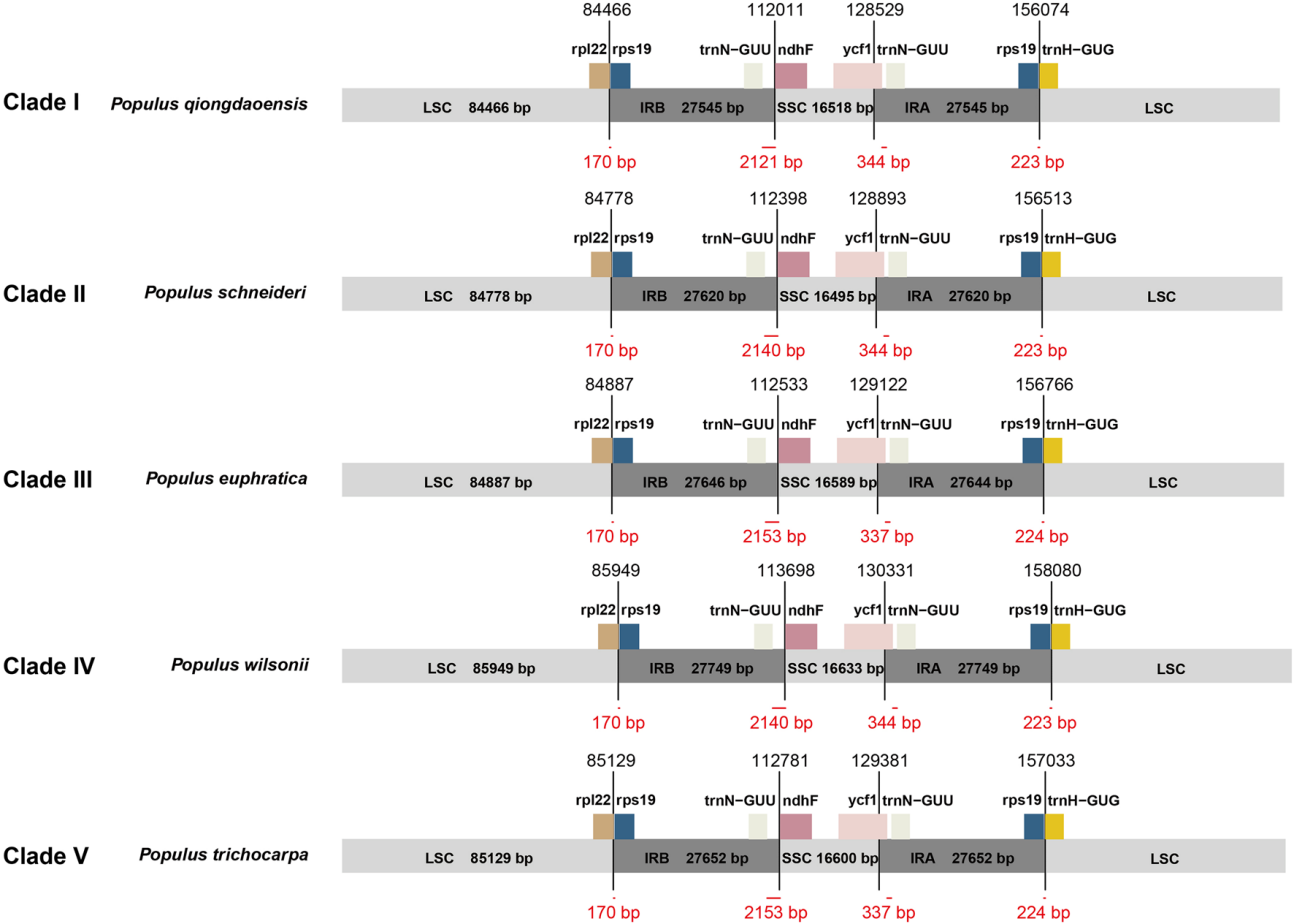
Comparison of junctions between the LSC, SSC, and IR regions among one exemplar species from each clade. Figure is not to scale. (LSC Large single-copy, SSC Small single-copy, IR inverted repeat).

Like nearly all land plants the chloroplast genomes of *Populus* are divided into four parts^17^, the large single copy region (LSC) and the small single copy region (SSC), separated by two inverted repeat regions (IRA and IRB, Fig. 7). Differences in the boundary of these four parts are measured by the distance (in nucleotides) of adjacent genes to the junctions and can, in some cases, provide phylogenetic signal in distinguishing lineages^54, 55^. In *Populus*, the genes adjacent to the junction boundaries are as follows: LSC-IRB with genes *rpl22-rps19*; IRB-SSC with genes *trnN-ndhF*; SSC-IRA straddled by *ycf1*; and IRA-LSC with genes *rps19-trnH*. To determine the degree to which differences in junction boundaries are heterogeneous across *Populus*, we chose eleven species (two from each clade and an outgroup) to compare boundary positioning. The distance of *rps19* was consistent across all species at 170 bp from the LSC-IRB boundary. The position of *rps19* was essentially constant at 223 or 224 bp from the IRA-LSC junction as was the position of *trnN*-GUU at either 337 bp or 344 bp from the boundary of SSC-IRA. The most variable junction boundary was that of IRB-SSC with *trnN* from 2121 to 2153 bp from the junction boundary. As such this junction boundary is the most variable of the four and is similar to other chloroplast genomes in this regard^30, 46^.

### Codon usage in *Populus* chloroplast genes

The relative synonymous codon usage (RSCU) is a ratio value of the frequency of the target codon to the expected frequency of that codon. If the RSCU is larger than 1, it indicates that the target codon is used more frequently than expected. A value smaller than 1 indicates that the codon is used less frequently than expected^56–58^. Using the RSCU value as a basis of comparison, we selected one species from each of the five clades (Fig. 2) to compare the distribution and frequency of codon usage (Figure S2).

From these comparisons (Figure S2), 64 types of codons encoding 20 amino acids and one stop codon were detected in all five species sampled. The distribution and frequency of those codons was very similar across *Populus*. Among the reported codons, Ter-UGA (one type of three stop codons but does not code for an amino acid) had the lowest RSCU value in all five species. The Tyr-UAC encoding the tyrosine amino acid had the lowest RSCU value of all 20 amino acids, and the Gly-GGA encoding the glycine amino acid had the highest RSCU value. Methionine was the least frequent amino acid, and glycine (Gly), arginine (Arg) and serine (Ser) were the most frequent amino acids in all species. The most frequent amino acids in *Populus* differed from the *Lagerstroemia* species where leucine, arginine and serine were the most abundant^59^. As such codon usage may be mostly conserved at the genus level but vary when compared to more distant lineages. These analyses also revealed that one-half of the codons were used more frequently than expected with an RSCU value > 1, of which all of them ended with A/U. These codon usage patterns were similar to those reported in other angiosperms, possibly due to the high proportion of A/T nucleotides present in chloroplast genomes^60^.

### Clade ages in *Populus*

**Figure 8:**
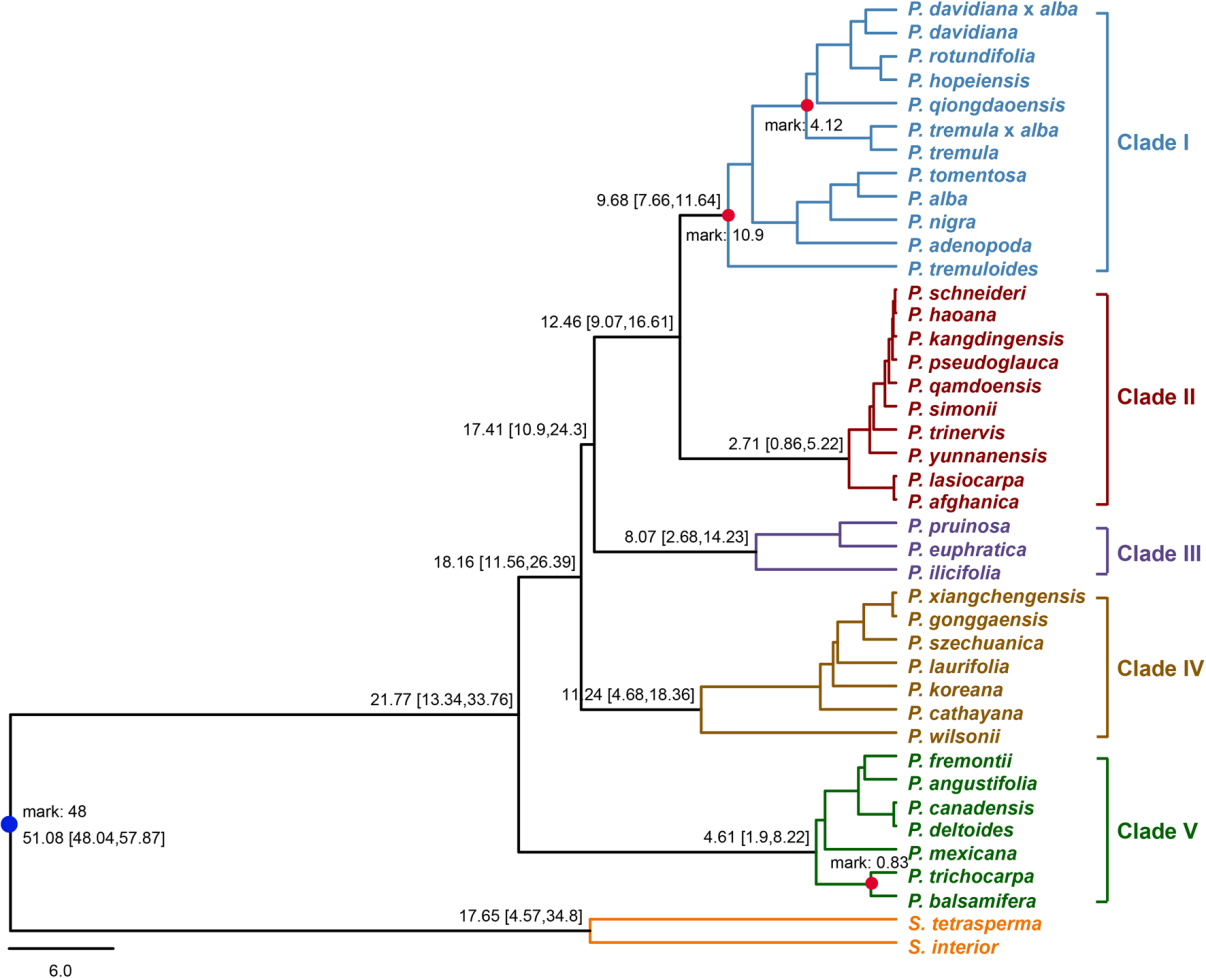
Ages of selected lineages of *Populus* inferred from 76 chloroplast genes. The blue dot indicates the earliest known fossils of ancestral *Populus* species^44^, and red dots represent calibrations used from the TimeTree website (http://www.timetree.org).

Another important application of chloroplast genome data is in estimating clade ages^24, 25, 31^ especially in genera like *Populus* where rampant hybridization can obscure signals of more ancient divergence^39^. Based on a matrix of 76 chloroplast coding genes and the earliest recognizable fossils of ancestral *Populus* species (~ 48 MYA), we inferred the age of clades in *Populus* by employing BEAST^61^ (Fig. 8). From this analysis the median age of each major clade was 9.68 (7.66–11.64 MYA) for clade I, 2.71 (0.86–5.22 MYA) for clade II, 8.07 (2.68–14.23 MYA) for clade III, 11.24 (4.68–18.36 MYA) for clade IV, and 4.61 (1.90–8.22 MYA) for clade V. Ages for each clade varied considerably suggesting possible differences in evolutionary processes such as varying degrees of lineage sorting or adaptive radiation in regard to chloroplasts^39^.

### Chloroplast genome diversity in populations of *P. euphratica*

**Figure 9:**
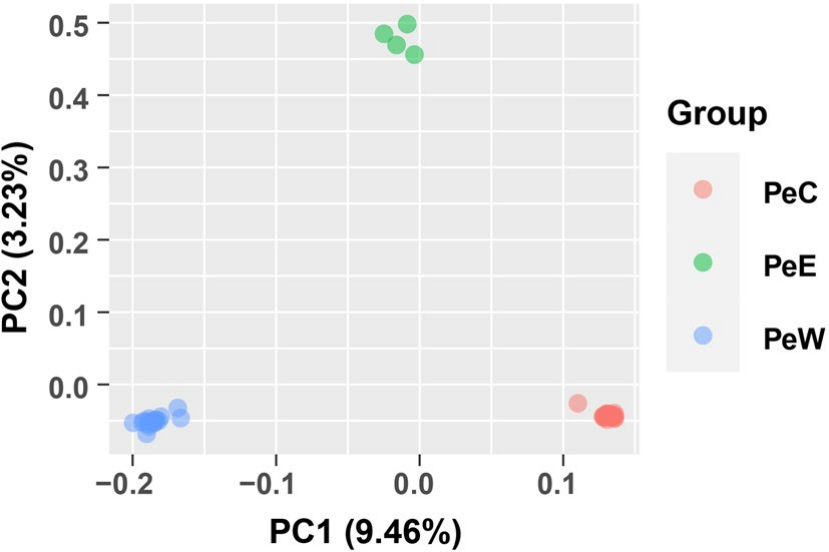
The principal component analysis (PCA) plot of chloroplast SNP data for 45 sampled individuals. Color coding reflects different geographic collection sites.

Before the development of NGS technologies, whole chloroplast genome resequencing for SNP discovery and population genomics was effectively impossible due to cost and efficiency constraints. Before whole chloroplast genomics a small number of chloroplast loci (*rbcL*, *matK*, *trnL-F*, etc.) were employed that provided limited resolution at the population level^28^. Because the chloroplast is non-recombinant and uniparentally inherited it can provide information on population divergence that can be lost in nuclear markers where interspecific introgression and repeated backcrossing can obscure signals of past divergence^17, 39^. To assess the utility of whole chloroplast genome data for population genomics we reanalyzed the nuclear genome resequencing data set of *P. euphratica* from Ma et al.^62^ to include only chloroplast SNPs which were not previous employed in their analyses. Principle coordinate analyses (PCA) and frequency-based population structure analyses were employed to assess divergence among individuals from across the range of *P. euphratica*. (Fig. 9). From these analyses it is clear that whole chloroplast derived SNPs provide adequate signal to identify divergent lineages of *P. euphratica*. The results using just the chloroplast SNPs largely reflect the results obtained by Ma et al.^62^ but provide improved resolution (PCA) given the absence of recombination from recent backcrossing.

To better understand the distribution of SNPs across the chloroplast genomes of *P. euphratica*, the number of SNPs in each 500-bp window were calculated (Figure S3). From this analysis it is clear that nucleotide variability at the population level is localized to the two single copy regions, as no SNPs (occurring in more than one individual) were found in the IR regions. High mutation rates in the single copy regions at the population level are also found at the genus level (Fig. 5) suggesting that regions used to delimit species may also have utility for population level analyses. More work is needed to understand how population level polymorphisms differ from species level polymorphisms in chloroplasts and how these polymorphisms (e.g., SNPs versus large insertions/deletions) could be used in population/species coalescent analyses.

## Discussion

In this paper, numerous different analyses using whole chloroplast genomes were conducted to demonstrate the utility and simplicity of this type of molecular data in making inferences regarding systematics and evolution. We chose the genus *Populus* because it is a model study system with nearly complete sampling of whole chloroplast genomes across all species as well as an example of population level whole chloroplast genome resequencing. *Populus* was expected to provide a suitable example of what to expect in regard to phylogenetic resolution, dating, and population genetics in other genera once they have been more comprehensively sequenced. Because reticulate evolution appears to be commonplace among numerous lineages of *Populus*^36^, the chloroplast genome may be especially relevant in detecting signals of divergence lost to abundant introgression or at least provide a comparative genome for finding and cross-dating divergent islands in the nuclear genome^62^. As such chloroplast genome data should be applied to other long-lived frequently hybridizing plant lineages to see if similar patterns are common. In addition, differences in clade ages, species richness, and the branch lengths of *Populus* subclades provide a comparative basis for testing hypothesis on adaptive radiation and the evolution of chloroplast genomes (Fig. 8).

The large-scale structural features of chloroplast genomes were found to be largely conserved across species of *Populus.* For instance, as has been found in other tree genera such as *Pterocarpus*^46^ junction boundaries do not vary by more than 50 bp. As such at the genus level while chloroplast genome size can vary by 3.5 Kb, positioning of the genes adjacent to the junction boundaries are strongly conserved at the genus level but less so at the family level. Furthermore, the length from gene to gene at junction boundaries does not appear to have a phylogenetic signal with the length from *trnN*-GUU to *ndhF* 2140 bp in early diverging *P. wilsonii* (clade IV), 2153 bp in later diverging *P euphratica*, (clade III), and back to 2140 bp in yet later diverging *P. schneideri* (clade II). As such junction boundaries in *Populus* are convergent in respect to total nucleotide length as compared to the phylogeny based on protein coding gene alignments (Fig. 2). This suggests that junction boundary distances may serve to delimit genera but not subclades within genera. In a similar manner the number of poly A/T repeats (and to some degree codon usage) appear to differ with little to no overlap between genera but not within genera. For instance, the number of poly A/T repeats at the 10 bp cutoff in *Populus* varies between 98 and 118 while in the sister genus *Salix* the number does not exceed 90. Interestingly intergeneric viable offspring are far less common than interspecific viable offspring in plants which may in part be linked to the pattern of conserved features at the genus level but not above^63^.

Directional selection on functional genes in chloroplasts is relatively uncommon across very long periods of time indicating that purifying selection maintains functional continuity in chloroplast genes^64^. However, chloroplast genomic studies are finding that generally a handful of genes within the genome have evolved via positive selection^64–66^. Our findings for *Populus* also found several genes under positive selection when comparing subclades within the genus. Given these findings more detailed and widespread studies need to be conducted to document and improve our understanding of positive selection in the evolution chloroplast genes. Genus and family level studies that integrate gene expression data and rate evolution of nuclear-chloroplast-interacting genes may clarify why different chloroplast genes have undergone different modes and rates of selection.

While the chloroplast genome is generally viewed as highly conserved and little changed, our and other findings^2^, suggest that even at the population level the chloroplast genomes contain ample SNPs for resolving population structure. More work is needed to place these population level differences in context through intensively sampled sister-species comparative studies and data partitioning to understand how these changes may lead to functional changes between populations including the evolution of cytonuclear conflict in hybrids^67^. Comparative studies with *Populus* might prove especially useful in understanding cytonuclear conflict as strong reproductive barriers between lineages do not appear to exist given extensive evidence of past natural introgression^36, 39, 62^.

## Conclusions

Given the results of this study the use chloroplast genomes for phylogenomics, population genomics, and phylogeography should be encouraged. However as with the over 420 complete angiosperm nuclear genomes (https://www.plabipd.de) issues regarding the completeness of chloroplast genomes remains. In this study we found several examples of incomplete (seven of the 39 genomes contained Ns) and incorrectly annotated, as well as potentially mislabeled (in regard to species identification in GenBank) chloroplast genomes in the genus *Populus* that has been intensively sampled and frequently studied. Because chloroplast genome sequencing and assembly is simpler and less expensive, as well as the fact that more chloroplast genomes have been sequenced for reference, correcting errors in chloroplast genomes is much easier than with massive nuclear genomes. As such not only should efforts be taken to correct errors in previously published chloroplast genomes but groups with less sampling such as algae, bryophytes, lycophytes, and ferns should be more intensively sampled such that a clearer understanding of chloroplast evolution can be achieved. As more chloroplast genomes are sequenced and published the analytical and applied uses will expand and understanding of evolutionary processes clarified. Herein we have outlined many of the analyses that can be conducted at present and what to expect from a genus with nearly comprehensive species sampling.

## Materials and methods

### Genome annotation

The 39 complete chloroplast genomes of *Populus* and two *Salix* species were downloaded from the NCBI GenBank, from which *P. schneideri* was used as a reference, with all genes in these two genera delimited manually to provide a complete reference template. The re-annotation of all species was then executed using Plastid Genome Annotator (https://github.com/quxiaojian/PGA).

### Genome nucleotide diversity

Analyses of genome sequence diversity was done using mVISTA (http://genome.lbl.gov/vista/mvista/submit.shtml) to compare five *Populus* species with the Shuffle-LAGAN alignment program with the *P. trichocarpa* cp genome used as a reference. All 41 cp genomes were split into several parts based on annotation files, and the overall consistency score of each part was calculated with multiple sequence alignment tools using T-Coffee^53^ in default mode.

### Phylogenetic analysis

The whole cp genome sequence alignment of 39 *Populus* species and two outgroup *Salix* species was generated with the MAFFT v7.464^68^ software, and TrimAL v1.4 (http://trimal.cgenomics.org/) used to trim poorly aligned positions. The longest CDS sequences of 76 protein-coding genes were extracted from each genome according to the annotation files, and also aligned using MAFFT. The PAL2NAL v14^69^ program was used converted the multiple sequence alignment of DNA and its corresponding proteins sequences into a codon alignment. For the amino acid matrix, protein sequences derived from CDS sequence followed the same treatment of whole cp genome sequence alignment. Thereafter, nucleotide and amino acid sequence alignments of 76 protein-coding genes were connected together. These three data sets (complete genome DNA sequences, all CDS DNA sequences, and amino acid sequence from all CDS DNA) of *Populus* species cp genomes were used to reconstruct the phylogenetic tree using IQ-TREE v2.0^70^ with 1000 ultrafast bootstrap replicates to assess clade support with iTOL (https://itol.embl.de) used for tree visualization.

### Repeat and SSR detection

Four repeat types in *Populus* cp genomes, F (forward), P (palindrome), R (reverse), and C (complement) were identified using REPuter (https://bibiserv.cebitec.uni-bielefeld.de/reputer) with default settings. Simple sequence repeats (SSRs) were detected using the Perl script MISA^71^, with 10, 6, 5, 5, 5, and 5 repeat units set for mono-, di-, tri-, tetra-, penta-, and hexa-motif microsatellites set as the minimum threshold for detection respectively.

### Codon usage and dN/dS analyses

The CodonW v1.4.4 software was employed to assess codon distribution on the basis of relative synonymous codon usage (RSCU) ratio^72^. CODEML in PAML v4.9^73^ was used to estimate the nonsynonymous (dN) and synonymous substitutions (dS) and the ratio of nonsynonymous to synonymous nucleotide substitutions (dN/dS) for each branch based on the above phylogenetic tree.

### Junction boundary analysis

The distance between the adjacent genes and the four junctions were used to estimate the variation in cp genomes.

### Clade age estimates

To estimate the ages for major clades in *Populus*, the combined nucleotide matrix of 76 protein-coding genes and the topology derived from IQ-TREE were used. The program Beast v2.6.2^61^ was used to estimate the age of different *Populus* lineages using the GTR model and the earliest fossils of ancestral *Populus* species, 48 MYA^44^ used for calibration. Calibration points from the website TimeTree (http://www.timetree.org) were also employed. They included divergence times between *P. balsamifera* and *P. trichocarpa* of 0.83 MYA, *P. tremula* and *P. davidiana* at 4.12 MYA, and the calibrated divergence time for clade I of 10.9 Mya was used.

### Chloroplast genome and population structure analysis of *Populus euphratica*

Illumina generated data from 102 individuals of *P. euphratica* was downloaded from NCBI under the project PRJNA380894 and initially processed with Fastp^74^ to deal with adapter low quality reads. After that, reads were mapped to the reference cp genome of *P. euphratica* using BWA-MEM^75^, and GATK v4.0.12^76^ HaplotypeCaller was employed for SNP calling followed by VariantFiltration with filter parameters ‘QUAL < 60, QD < 20.0, FS > 10.0, MQ < 30.0, MQRankSum <  − 1.65 and ReadPosRankSum <  − 8.0′. VCFtools v0.1.16^77^ and PIC_CALC (https://github.com/luansheng/PIC_CALC) were used for PIC analysis. The 3 *P. euphratica* lineages PeC (24 individuals), PeE (4), and PeW (17) were selected for principal components analysis (PCA) with Plink v1.9^78^, which contained the detailed location information.

## Supplementary Information

Supplementary Information 1.

Supplementary Information 2.

Supplementary Information 3.

Supplementary Information 4.
