# Supplementary material for: Chloroplast genomes in Populus (Salicaceae): comparisons from an intensively sampled genus reveal dynamic patterns of evolution
Source: Sci Rep. 2021 May 4;11:9471. doi: 10.1038/s41598-021-88160-4 (PMC8096831; doi:10.1038/s41598-021-88160-4)
Supplement: Supplementary file 1 — Supplementary Information 1. [file 41598_2021_88160_MOESM1_ESM.docx]

**Supplementary information**


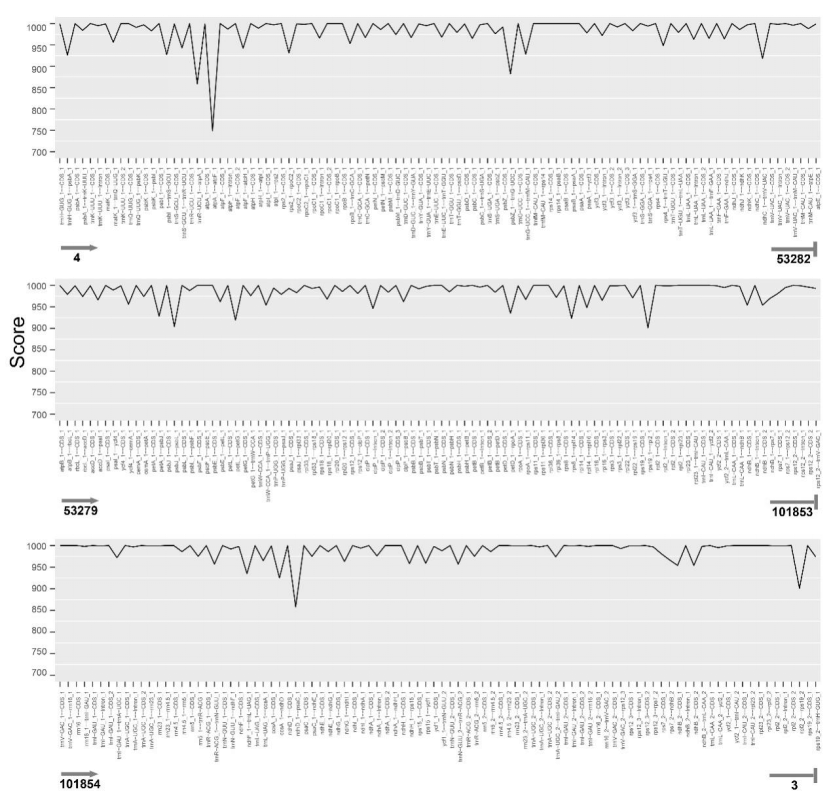


**Figure S1**. Sequence identity among coding and non-coding regions based on the alignment from 39 *Populus* species. T-Coffee was used to calculate the score of identity.


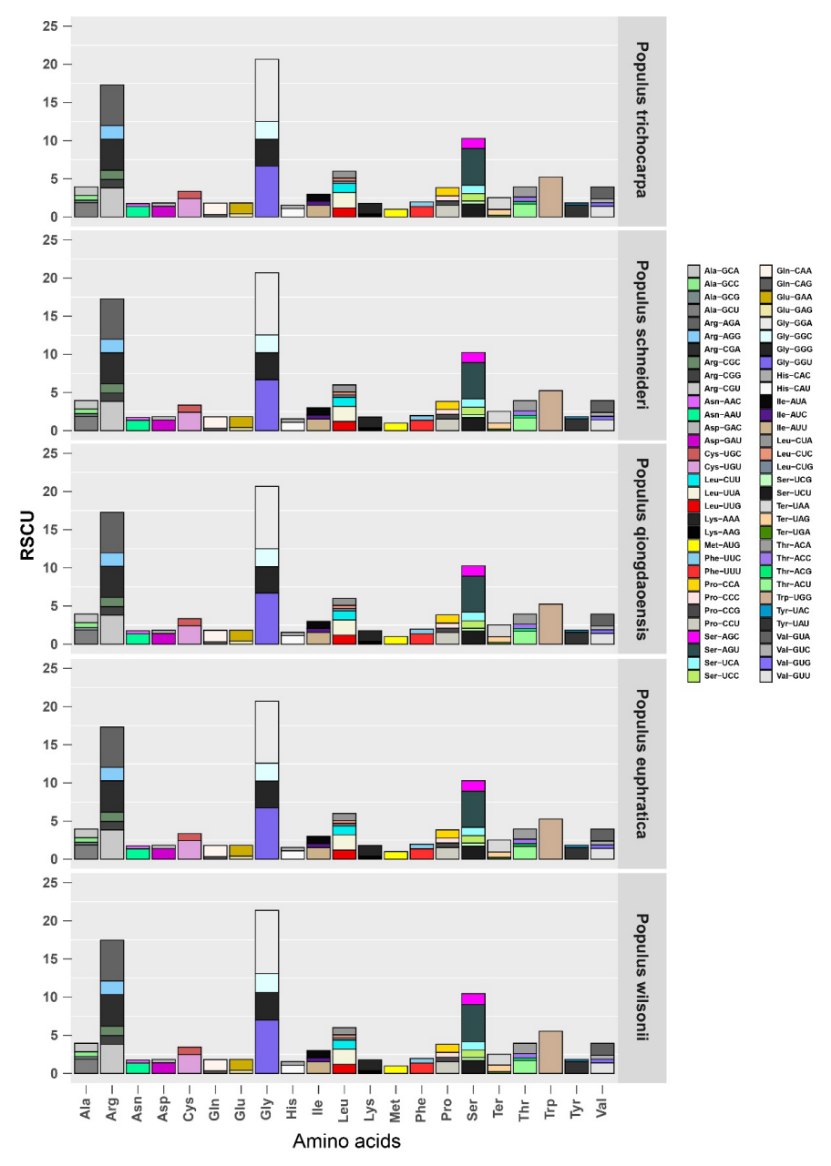


**Figure S2**. Codon content of 21 amino acids and stop codons for 76 coding genes of five *Populus* species. Color of the histogram corresponds to the color of codons in the legend.


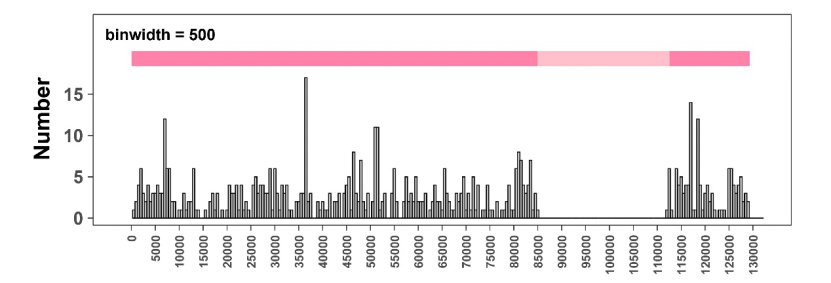


**Figure S3**. The distribution of SNPs across the chloroplast genome from 102 individuals of *P. euphratica*. The two dark pink regions refer to the large and small signal copy regions. The light pink refers to the inverted repeat region. SNPs that were only present in a single individual were removed.
