# Supplementary material for: Chloroplast genomes in Populus (Salicaceae): comparisons from an intensively sampled genus reveal dynamic patterns of evolution
Source: Sci Rep. 2021 May 4;11:9471. doi: 10.1038/s41598-021-88160-4 (PMC8096831; doi:10.1038/s41598-021-88160-4)
Supplement: Supplementary file 2 — Supplementary Information 2. [file 41598_2021_88160_MOESM2_ESM.docx]

**Table S1**. The distribution of finished chloroplast genome and the top 10 groups in different classification level.

**Table S2**. The reannotated gene content in all 39 chloroplast genomes of *Populus*.
